# Characteristics and outcomes of patients with multiple myeloma at the Uganda Cancer Institute

**DOI:** 10.4314/ahs.v21i1.11

**Published:** 2021-03

**Authors:** Clement D Okello, Yusuf Mulumba, Abrahams Omoding, Henry Ddungu, Kristen Welch, Cheryl L Thompson, Andrew J Cowan, Matthew M Cooney, Jackson Orem

**Affiliations:** 1 Uganda Cancer Institute; 2 Case Western Reserve University; 3 University of Washington, Seattle, WA

**Keywords:** Multiple myeloma, Uganda cancer institute

## Abstract

**Purpose:**

Data on multiple myeloma (MM) in sub-Sahara Africa is scarce. In Uganda, there is a progressively increasing incidence of MM over the years.

**Methods:**

We performed a retrospective study on 217 patients with MM at the UCI using purposive sampling method. The objectives of the study were to determine the clinical characteristics, treatment outcomes, 5 year overall survival and predictors of survival of patients with MM at the UCI from 01 January 2008 to 31 December 2012.

**Results:**

There were 119 (54.8%) males; the mean(SD) age of the study population at presentation was 59(12.8) years; 183(84.3%) patients presented with bone pain, and 135 (61.9%) had skeletal pathology; 186(85.3%) were HIV negative, and 152(70%) had Durie-Salmon stage III. The median overall survival was 2.5 years, (95% CI, 0.393–0.595); factors significantly associated with worse survival were Durie-Salmon stage III disease, HR=5.9, 95% CI (1.61 – 21.74; P=0.007) and LDH >225 U/L HR=3.3, 95% CI (0.57 – 5.92; P=0.029).

**Conclusion:**

Most patients with multiple myeloma at the UCI were diagnosed at a relatively young age, presented with late stage disease and bone pain, and had a shorter survival time. Factors associated with worse survival were Durie-Salmon stage III and LDH >225 U/L.

## Introduction

Multiple myeloma (MM) is the second most common hematologic malignancy in the United States^1^–^3^ and it is the most common hematologic malignancy among African Americans in the US^4^. The incidence in African Americans and blacks from Africa is two to three times that in whites^5^–^7^. In contrast, the risk is lower in Asians from Japan and in Mexicans^6^, ^8^. Myeloma is slightly more common in males thn females^9^ with a median age at diagnosis of 66 years; only 10% and 2% of patients are younger than 50 and 40 years, respectively^5^. Other risk factors for MM include positive family history^10^. Survival improvement of patients with MM is much less pronounced among African Americans than whites^11^. Data on multiple myeloma and other plasma cell neoplasms for the large population living in sub-Sahara Africa is scarce. The few available studies include one from the 1970s which demonstrated age adjusted incidence rates of 7.47 per 100,000 in black males and 5.11 per 100,000 in black females^12^. In this study, the median age of clinical presentation in the black patients was 52 years, 10 years younger than the median age for the white patients^12^. Small studies in Nigeria and Kenya reported the mean age at diagnosis of 52 years in the 1970s and 1980s^13^,^14^, and 62 in 2014 in Nigeria^15^, with the longest duration of survival of less than 5.5 years in patients treated with melphalan and prednisolone with or without thalidomide.

In Uganda, MM has been demonstrated to show a progressively increasing incidence rate (per 100,000) of 0.4, 0.7, and 1.9 among males and 0.5, 0.9 and 1.7 among females for the periods of 1991–1995, 1996–2001, and 2002–2006 respectively^16^. There is no clear explanation for this trend, but it is reasonable to suggest that there is relatively better diagnostic capability and increased health workers force in Uganda currently than in the past. A report by the World Health Organizatin in 2014 indicates that multiple myeloma was responsible for mortality in 6.0% of males and 5.0% of females among 9,100 and 9,000 cancer deaths respectively^17^.

Patient characteristics, such as race and environmental factors, are important determinants in the aetiology and prognosis of MM^11^,^18^. Currently, most data on MM have been reported by studies performed outside Africa; these data may not entirely apply to the African population. Therefore, we performed a retrospective study on 217 patients with multiple myeloma at the Uganda Cancer Institute to determine their clinical characteristics, treatment outcomes, and 5 year overall survival ad predictors of survival.

## Methods

### Study design and study setting

This was a retrospective observational cohort study based on chart review conducted at the Uganda Cancer Institute (UCI). UCI is the only tertiary cancer treatment facility in Uganda. It also serves patients from some of its neighbouring countries including the Democratic Republic of Congo, South Sudan, Kenya, Tanzania, Burundi and Rwanda.

### Eligibility criteria

Data was abstracted from 217 charts of patients meeting the International Myeloma Working Group (IMWG) Diagnostic Criteria for multiple myeloma(19) and were seen at the UCI from 01 January 2008 to 31 December 2012 (5 year period) using homogenous purposive sampling method. No charts were excluded.

### Data collection

Eligible charts were identified by the UCI Records Officer with the help of a study assistant. Data was abstracted from charts using a standard data collection tool. Data on treatment response was reported as documented on the patients' charts according to the IMWG response criteria. Completed data collection tool was checked for completeness and accuracy by the principal investigator. Data was then coded, and double entered into a computer using Epidata version 3.1 (Epidata association, Denmark). After validation, data was exported to STATA Version 14 (StataCorp, USA) for analysis. Study approvals were obtained from the Uganda Cancer Institute Research Ethics Committee (UCIREC) and the Uganda National Council for Science and Technology (UNCST). Waiver of consent was obtained since this was a retrospective chart review study.

### Statistical analysis

The five (5) year overall survival was defined from the time of diagnosis of MM. Cox proportional hazard model was used to evaluate the association between patient characteristics and OS at bivariate and multivariate analyses starting with known factors associated with survival and then others. Patients who were lost to follow up were included in the analysis and were censored on the last recorded date of review at the UCI. Hazard ratios and 95% confidence intervals were generated. Data analysis was performed using STATA Version 14 (StataCorp, USA). Statistical significance was set at p<0.05 (two-sided).

## Results

### Baseline clinical characteristics

A total of 217 patients with a diagnosis of MM were identified. There were more males (n=119, 55%) than females (n=98, 45%). The mean (SD) age at presentation was 59 (12.8) years. Of the 111 patients who had documented weight and height, the median(IQR) Body Mass Index (BMI) was 22.3(+ 6.1) kg/m2; of the 118 patients who had documented LDH, 45(38%) had LDH <225 U/L and 73(62%) had LDH >225 U/L. Majority of patients (n=183, 84%) presented with bone pain, were HIV negative (n=186, 85%), and had Durie-Salmon stage III disease (n=152, 70%). The majority of patients did not have records of serum beta-2 microglobulin. All patients had morphological evidence of plasmacytosis of bone marrow sample or other available tissues and results for serum protein electrophoresis (SPEP) but only 25 (9.9%) patients had immunofixation done. Of these, a majority (n=18, 72%) had IgG kappa while the rest (n=7, 28%) had IgA lambda. A majority of patients (n=111, 69%) presented with lytic bone lesions or evidence of fracture. Table 1 illustrates the baseline characteristics.

**Table 1:** Baseline demographic and clinical characteristics

Characteristic	Value
**Age (years), mean(SD)**	59(12.8)
**Sex, n(%)**	
Male	119(55)
Female	98(45)
**Symptom at presentation, n(%)**	
With pain	183(84)
No pain	34(16)
**HIV sero-status, n(%)**	
Negative	185(86)
Positive	12(6)
Unknown	20(9)
**Durie Salmon Stage, n(%)**	
I	27(12)
II	32(15)
III	152(70)
Unknown	6(3)
**Monoclonal type, n(%)**	
IgG kappa	18(72)
IgA Lambda	7(28)
**LDH (U/L), n(%)**	
≤225	45(38)
>225	73(62)
**Serum creatinine (umol/L), (n=186);** Median(IQR)	102.1(68 – 228)
**Albumin (g/L), (n=156);** Mean(SD)	31.8 (7.8)
**Calcium (mmol/L), (n=121);** Mean(SD)	2.4 (0.7)

### Response to treatment

Of the 155 patients who received chemotherapy, the majority, 93 (60%) received Melphalan and Prednisolone (MP); followed in descending order by Melphalan, Prednisolone and Thalidomide (MPT), 21 (13.6%); Vincristine, Doxorubicin and Dexamethasone (VAD), 18 (11.6%); Bortezomib based, 10 (6.5%); and Thalidomide and Dexamethasone (TD), 7 (4.5%). The median (IQR) number of chemotherapy cycles received were 6 (5) for TD, 5 (7) for MP, 4 (5) for VAD, 3 (4) for Bortezomib, 2 (5) for MPT, and 2 (5) for other. The overall treatment response rate was highest for TD (57.1%, 95% CI, 15.0 – 90.9), followed in descending order by Bortezomib (37.5%, 95% CI, 8.7 – 79.2), VAD (29.4, 95% CI, 11.5 – 57.1), MP (25.0%, 95% CI, 17.1 – 35.0) and MPT (9.5%, 95% CI, 2.1 – 34.0). CR and PR also follow similar patterns, Table 2.

**Table 2:** Treatment response rates

	TD	Bortezomib	VAD	MP	MPT	Other
**ORR** (95% CI)	57.1 (15.0–90.9)	37.5 (8.7–79.2)	29.4 (11.5–57.1)	25.0 (17.1–35.0)	9.5 (2.1–34.0)	33.3 (4.2–85.1)
**CR** (95% CI)	57.1 (15.0–90.9)	25 (4.1–72.4)	17.6 (5.0–46.3)	21.7 (14.4–31.5)	9.5 (2.1–34.0)	33.3 (4.1–85.1)
**PR** (95% CI)	0	12.5 (0.9–68.1)	11.8 (2.5–40.9)	3.3 (1.0–9.8)	0	0
**PD** (95% CI)	0	0	0	3.3 (1.0–9.8)	4.7 (0.5–30.9)	0
**SD** (95% CI)	42.9 (9.1–85)	62.5 (20.8–91.3)	70.6 (42.9–88.5)	71.7 (61.5–80.1)	85.7 (2.1–34.0)	66.7 (14.9–95.8)
**Patients** n(%)	7(4.5)	10(6.5)	18(11.6)	93(60)	21(13.6)	6(3.9)

**Note:** CI: Confidence Interval; CR: Complete Response; MP: Melphalan and Prednisolone; MPT: Melphalan, Prednisolone and Thalidomide; ORR: Overall Response Rate; PR: Partial Response; TD: Thalidomide and Dexamethasone; VAD: Vincristine, Doxorubicin and Dexamethasone.

### Survival

The median overall survival for all patients in the study was 2.5 years (95% CI, 0.39–0.60). The median survival for patients in Durie-Salmon stage III was 1.7 years (95% CI, 0.37–0.59) (Figure 1), but was not reached for patients in stage I and stage II (Figure 2).

**Figure 1:**
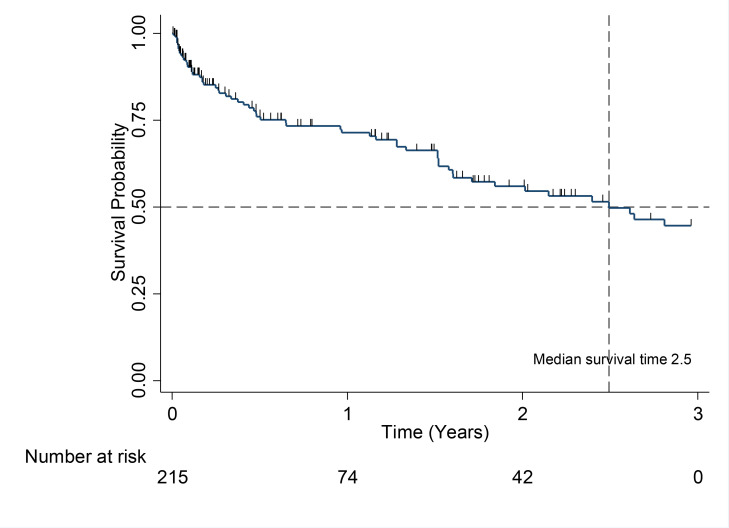
Kaplan-Meier Overall survival estimate

**Figure 1:**
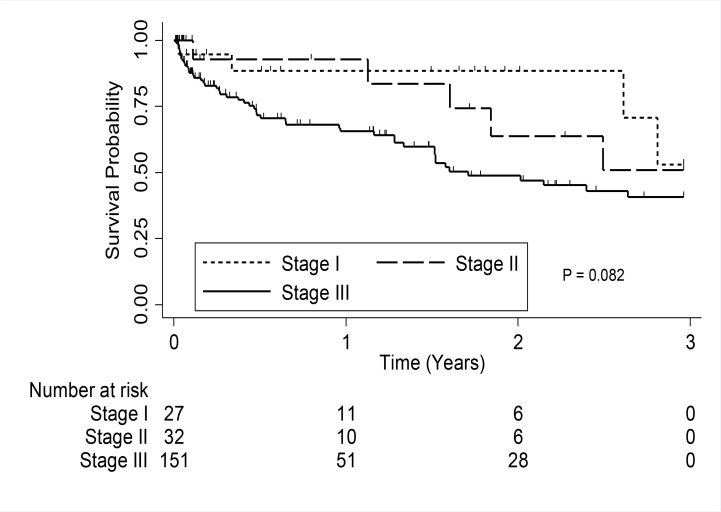
Kaplan-Meier survival estimate by stage of Multiple Myeloma

At multivariable analysis, factors that were found to be significantly associated with survival were Durie-Salmon stage III disease HR=5.9, (95% CI, 1.61 – 21.74; P=0.01) and LDH >225 U/L HR=3.3 (95% CI, 0.57 – 5.92; P=0.03). BMI and HIV status were not significantly associated with survival (Table 3).

**Table 3:** Bivariable and multivariable analyses of survival

	Bivariable analysis		Multivariable analysis	

Factor	CHR (95%CI)	P-Value	AHR(95%CI)	P-Value
Age	1.0(0.99 – 1.03)	0.24	1.0(0.99 – 1.05)	0.12
Gender	0.7(0.41 – 1.14)	0.15	0.7(0.32 – 1.48)	0.34
Comorbidities	1.7(1.03 – 2.84)	0.04	1.7(0.79 – 3.80)	0.17
Disease stage				
I	1.0	.	1.0	.
II	1.4(0.42 – 4.66)	0.59	1.5(0.23 – 9.85)	0.68
III	2.6(0.99 – 6.74)	0.05	5.9(1.61 – 21.74)	0.01
BMI category				
Underweight	1.0	.	1.0	.
Normal	1.5(0.53 – 4.42)	0.44	1.1(0.27 – 4.83 )	0.86
Overweight	1.0(0.31 – 3.30)	0.10	0.6(0.10 – 3.30)	0.52
HIV test				
Negative	1.0	.	1.0	.
Positive	1.2(0.30 – 4.55)	0.83	0.5(0.07 – 2.82)	0.40
LDH				
≤ 225 U/L	1.0	.	1.0	.
>225 U/L	1.7(0.83 – 3.41)	0.15	3.3(1.13 – 9.50)	0.03
No treatment received	0.1(0.06 – 1.13)	<0.001	9.5(4.23 – 21.55)	<0.001

NB: AHR: Adjusted Hazard Ratio, CHR: Crude Hazard Ratio, CI: Confidence Interval

## Discussion

Multiple myeloma is a cytogenetically heterogeneous clonal plasma cell proliferative disorder^20^, ^21^ that generally reflects a chromosomal abnormality, with many translocations involving chromosomes 13 and 14^22^, ^23^. There are no established risk factors other than male gender, increasing age, African-American ethnicity, positive family history of cancers of haematopoietic and lymphoid tissues and monoclonal gammopathy of undetermined significance (MGUS)^24^, ^25^,^26^. Multiple myeloma has not yet been very well studied in the sub-Saharan Africa. To the best of our knowledge, our study is so far the largest of its kind to describe the clinical characteristics and outcomes of patients with MM in the sub-Saharan Africa.

Our data is consistent with the observation that MM is more prevalent in males^26^; however, compared with the global statistics which reports the mean age at diagnosis of MM as 66 27–29, our data depicts a younger mean(SD) age at diagnosis of 59 (12.8) years. In a series of 123 patients (49 black and 74 white) observed over 1971 to 1976 in Johannesburg, South Africa,^30^ the median age of clinical presentation in the black patients was 52 years, 10 years younger than the median age for the white patients. A recent report by Madu et al.^15^ in 2014 on a retrospective analysis of 32 patients diagnosed with multiple myeloma in Nigeria showed a median age at diagnosis of 62 years. Mukiibi et al. in 1988^31^ in a study on 75 patients with MM in Kenya, reported a mean age at presentation of 51 years with 66.7% presenting with bone pains. The younger age of presentation in African patients has been viewed to be due to the overall population age distribution^32^, however, genetic factors may also play a role.

Few patients in our study were tested for serum immunoglobulins. This test is not available to most patients at the UCI due to cost. Most patients (72%) who could afford the test had IgG kappa type MM, and the remaining had IgA. This finding is not very different from a study on Afro-Caribbean patients in the United States of America that observed 64% patients with IgG MM followed by 17% IgA MM. However, Schulman et al., 1980, in their study in south Africa reported a higher frequency of IgA myeloma in the black patients^30^.

A majority of patients (69.7%) in our study presented with late stage disease (Durie-Salmon stage III), symptomatic bone pain (84.4%) and radiographic evidence of bone disease (61.9%). In contrast, the study on Afro-Caribbean population reported only 30% of patients as presenting in Durie-Salmon stage III and 60% presenting with symptomatic bone pain. Presentation with late stage disease is not uncommon at the UCI and is not limited to only MM but to a wide number of cancers^33^.

A majority of patients (85.3%) in our study were HIV negative. An Australian cohort study reported an increased number of patients with multiple myeloma among persons with AIDS^34^. A similar observation was also reported in an earlier US and Puerto Rican AIDS registry study^35^. The prevalence of HIV among persons aged 15 – 49 years in Uganda was 5.7% in 2018^36^.

The median survival of patients with MM is approximately 5 – 7 years with survival varying depending on host factors, tumor burden (stage), biology (cytogenetic abnormalities), and response to therapy^37^–^39^. In contrast, the median survival of patients in our study of only 2.5 years, was much shorter than observed elsewhere. This may in part be due to presentation with late stage disease, but likely also related to treatment availability - in general, this cohort received treatments generally given for transplant ineligible patients, and generally sub-optimal compared to the currently recommended treatment for MM by international guidelines^40^. A majority of patients in our study were treated with MP, MPT and VAD, which are currently considered suboptimal induction regimens. Only a small number of patients were treated with Bortezomib-based chemotherapy. Moreover, the mean number of cycles received for most chemotherapy regimens were generally suboptimal^41^, ^42^. This could be due to the observed high loss to follow up.

The results of our study are consistent with the observation that advanced disease and high LDH confers an adverse prognosis in patients with MM^38^,^39^. However, we did not show patients' age as a predictor of survival. Increasing age has been shown to be associated with a shorter survival in many big studies^38^,^39^.

Our study had some limitations. The most important tumour factors that affect survival are genetic aberrations and gene expression profiles. Poor survival has been consistently associated with t(4;14) and 17p13 deletion^43^–^45^. However, patients in our study were not assessed for genetic aberrations due to resource limitations, therefore this data was unavailable to us. This study may not also have been powered to assess for other predictors of survival; moreover, a significant number of patients were lost to follow up, which is common in sub-Saharan Africa.

## Conclusion

our study is so far the largest on multiple myeloma done in the sub-Saharan African population. Our results show that most patients were diagnosed with MM at a relatively young age compared to global statistics, predominantly with late stage disease with mainly bone pain as a presenting symptom, and the majority were HIV negative. The patients in our cohort had a relatively shorter survival time. Factors associated with worse survival were late stage disease at presentation and high levels of LDH. Further studies should seek to further explain these unique observations in the sub-Saharan African population.

## Research support

Kleinman Family Research Fund & Uganda Cancer Institute.
